# Identification of key immune genes related to lymphatic metastasis of papillary thyroid cancer via bioinformatics analysis and experimental validation

**DOI:** 10.3389/fonc.2023.1181325

**Published:** 2023-05-18

**Authors:** Yang Yu, Xing Guo, Jian Chai, Zhuoyi Han, Yaming Ji, Jirui Sun, Huiqing Zhang

**Affiliations:** ^1^ Department of General Surgery, Baoding First Central Hospital, Baoding, Hebei, China; ^2^ Graduate School, Hebei Medical University, Shijiazhuang, Hebei, China; ^3^ Department of Oncology, Baoding First Central Hospital, Baoding, Hebei, China; ^4^ Department of Pathology, Baoding First Central Hospital, Baoding, Hebei, China; ^5^ Hebei Key Laboratory of Molecular Pathology and Early Diagnosis of Cancers, Baoding, Hebei, China; ^6^ Baoding Key Laboratory of Gastrointestinal Cancer Diagnosis and Treatment, Baoding, Hebei, China

**Keywords:** papillary thyroid carcinoma, lymphatic metastasis, immune infiltration, prediction model, machine learning

## Abstract

**Objective:**

The current research aimed to development and validation in signature immune genes for lymphatic metastasis in papillary thyroid cancer (PTC).

**Method:**

Weighted correlation network analysis (WGCNA) was performed to identify genes closely correlated with lymphatic metastasis in PTC from TCGA database. Information on immune-related genes (IRGs) was obtained from the ImmPort database. Crossover genes were used with the R package clusterProfiler for Gene Ontology and Kyoto Encyclopedia of Genes and Genomes enrichment. Key genes in the protein–protein interaction network of cross-targets were obtained using Cytoscape. Lasso and Random Forest (RF) models were utilized to identify pivotal genes. We constructed a nomogram based on the hub genes. The correlation between hub genes and immune cell infiltration was explored. We collected and assessed clinical samples *via* immunohistochemistry to detect the expression of hub genes.

**Result:**

In total, 122 IRGs were correlated with lymphatic metastases from PTC. There are 10 key IRGs in the protein–protein interaction network. Then, three hub genes including PTGS2, MET, and ICAM1 were established using the LASSO and RF models. The expression of these hub genes was upregulated in samples collected from patients with lymphatic metastases. The average area under the curve of the model reached 0.83 after a 10-fold and 200-time cross-validation, which had a good prediction ability. Immuno-infiltration analysis showed that the three hub genes were significantly positively correlated with resting dendritic cells and were negatively correlated with activated natural cells, monocytes, and eosinophils. Immunohistochemistry results revealed that lymph node metastasis samples had a higher expression of the three hub genes than non-metastasis samples.

**Conclusion:**

*Via* bioinformatics analysis and experimental validation, MET and ICAM1 were found to be upregulated in lymph node metastasis from papillary thyroid carcinoma. Further, the two hub genes were closely correlated with activated natural killer cells, monocytes, resting dendritic cells, and eosinophils. Therefore, these two genes may be novel molecular biomarkers and therapeutic targets in lymph node metastasis from papillary thyroid carcinoma.

## 1 Introduction

Thyroid carcinoma (THCA) is a common tumor of the female endocrine system. Its etiology is generally related to iodine intake, thyroiditis, radiation exposure, and other factors. Its main clinical characteristics include swelling of the neck, hoarseness, and difficulty swallowing. The histological types include follicular thyroid cancer (FTC), papillary thyroid cancer (PTC), anaplastic thyroid cancer (ATC), and medullary thyroid cancer (MTC). Among them, PTC is the most common type. In recent years, the actual detection rate of PTC has increased annually worldwide due to improvements in ultrasound technology and the popularity of fine-needle aspiration cytology of thyroid nodules. PTC originates from follicular epithelial cells derived from the endoderm. PTC has slow progression and a good prognosis. However, the incidence rate of lymph node metastasis in PTC remains high. In most types of tumors, lymphatic metastasis is closely correlated with poor prognosis, and it is an important indicator of tumor progression and a marker of worsening tumor stage. Therefore, early diagnosis of lymph node metastasis and active and effective treatment of patients are considered to be the key to improve the prognosis of patients with PTC complicated with lymph node metastasis.The appropriate biomarkers should not only monitor disease progression and response to therapy but also identify relapses in patients at high risk of infection (1–3). With the development of artificial intelligence and large data, machine learning is applied to the diagnosis and prognosis of THCA (4).

This research explores genes affecting lymphatic metastasis based on bioinformatics analysis and immunohistochemistry (IHC) to identify novel strategies for the clinical diagnosis and treatment of PTC.

## 2 Result

### 2.1 Identification of genes related to lymphatic metastasis *via* weighted correlation network analysis

**Figure 1:**
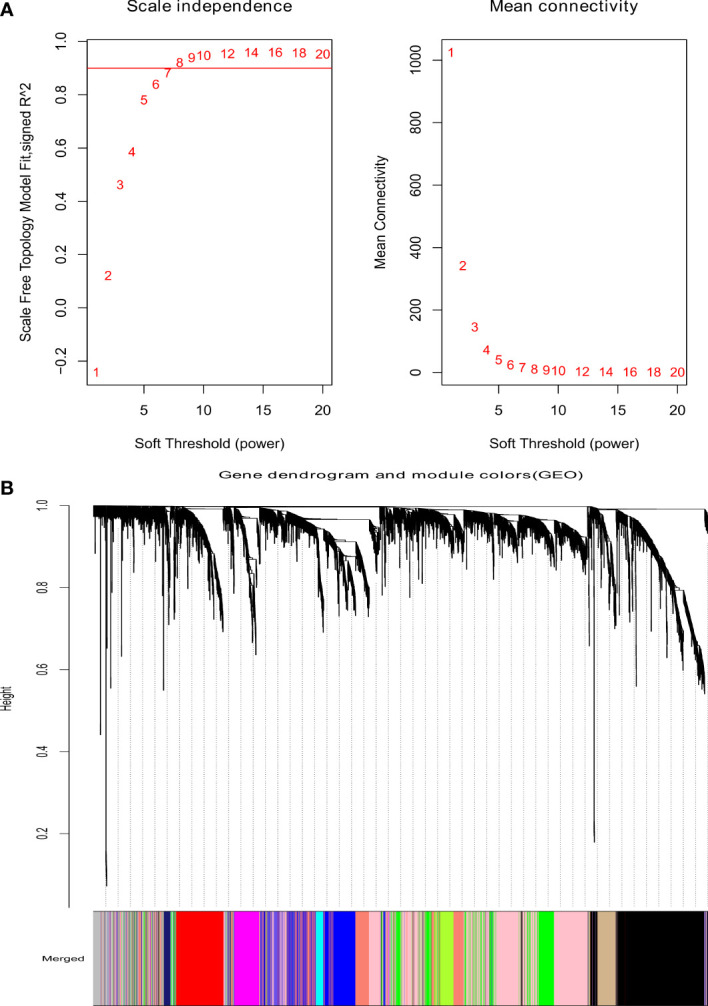
Analysis of weighted gene coexpression network. **(A)** The scale-free index for various soft-threshold powers (β) and the mean connectivity. **(B)** Dendrogram of genes clustered based on the measurement of dissimilarity.

**Figure 2:**
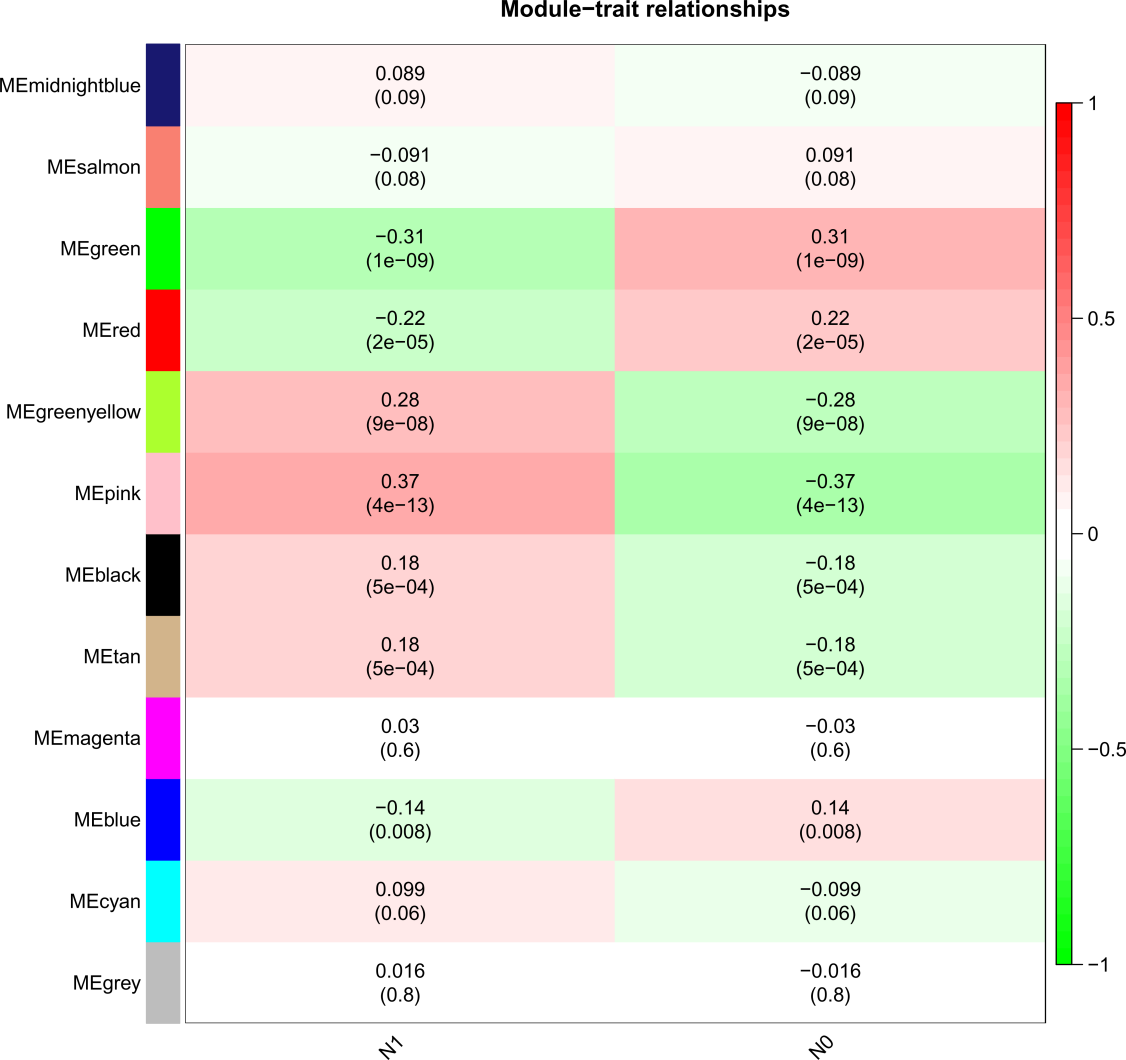
Heatmap of the correlation between the module eigengenes and Lymphatic metastasis of PTC.

As shown in **Figure 1A**, the optimal soft threshold β was set to 7 and the fitting coefficient R2 to 0.90, which is consistent with the topological distribution. **Figures 1B** show the gene clustering tree for hierarchical clustering analysis based on the difference in neighboring values. In this study, there were 12 modules, and the associations between them were evaluated. As shown in **Figure 2**, the black module was most closely correlated with lymphatic metastasis. This module was further analyzed, and it was found to contain 1271 genes.

### 2.2 Acquisition of the PPI network and key IRGs

**Figure 3:**
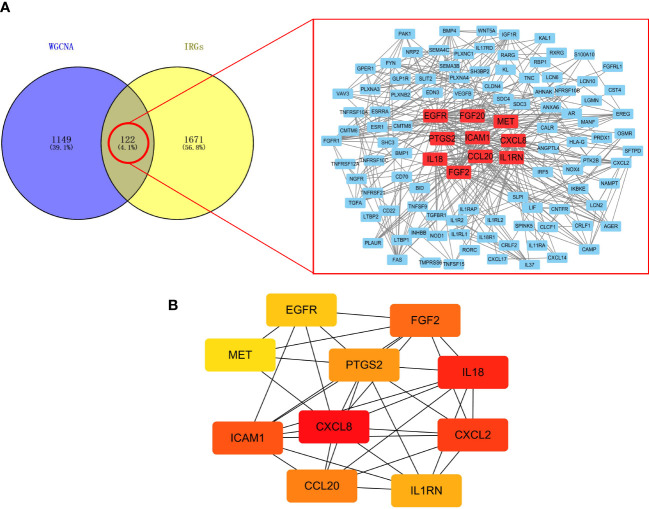
Screening of IRGs related to lymphatic metastasis in PTC. **(A)** PPI network. **(B)** Top 10 key IRGs.

There are 122 common genes in the black module and IRG, which are mapped as a PPI network (**Figure 3A**). The top 10 common IRGs, including CXCL8, CXCL2, PTGS2, and ICAM1, were obtained using the MCC algorithm in the cytoHubba application (**Figure 3B**).

### 2.3 GO and KEGG analysis

**Figure 4:**
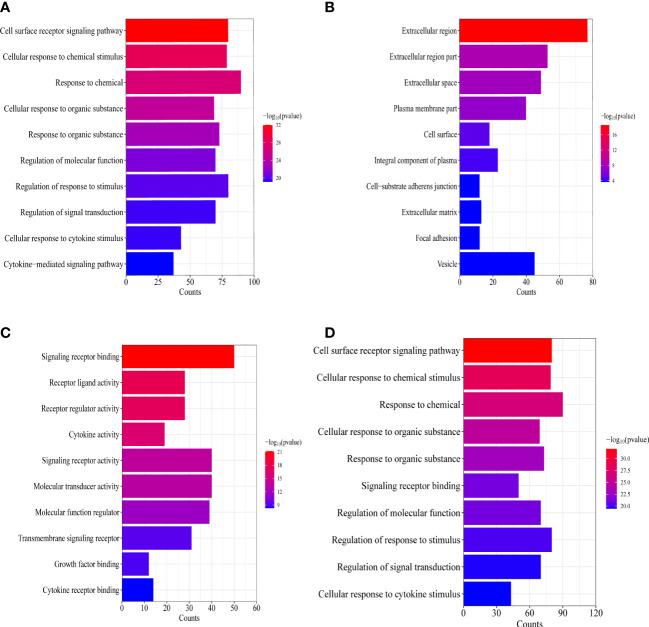
Enrichment analysis of the IRGs related to lymphatic metastasis in PTC. **(A)** Biological process analysis. **(B)** Cellular component. **(C)** Molecular function. **(D)** Kyoto Encyclopedia of Genes and Genomes.

To explain the molecular mechanisms of lymphatic metastasis related to immune genes, GO and KEGG analyses of 122 common genes were conducted. GO analysis revealed that lymphatic metastasis was functionally related to chemotaxis, signaling receptor complexes, receptor-ligand activity, and regulation of other biological functions (**Figures 4A–C**). KEGG analysis showed that cytokine–cytokine receptor interactions and natural killer (NK)-mediated cytotoxicity were involved in lymphatic metastasis (**Figure 4D**).

### 2.4 Establishment and evaluation of the prognostic model

**Figure 5:**
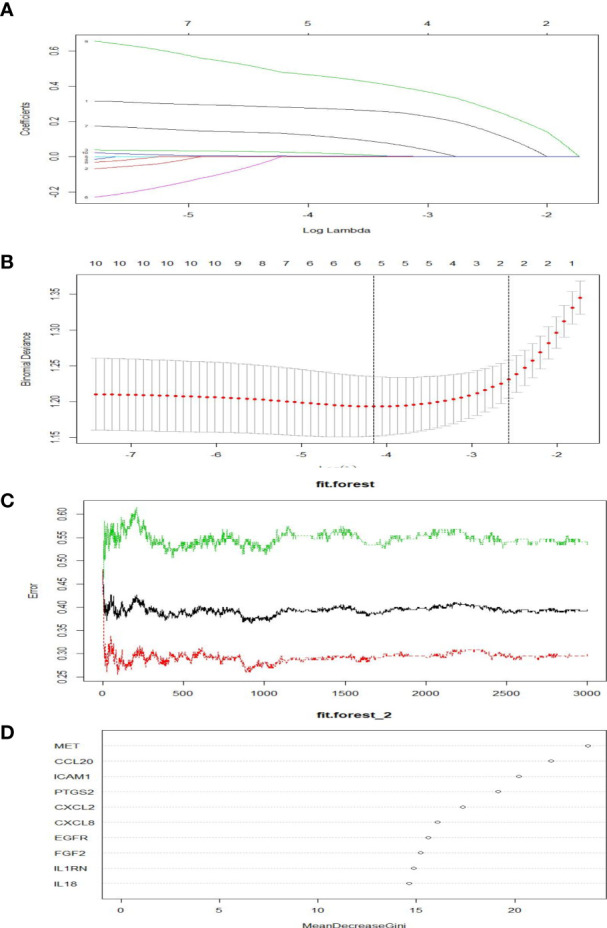
Screening of hubgens related to lymphatic metastasis in PTC. **(A, B)** Lasso regression. **(C, D)** Random forest classification.

**Figure 6:**
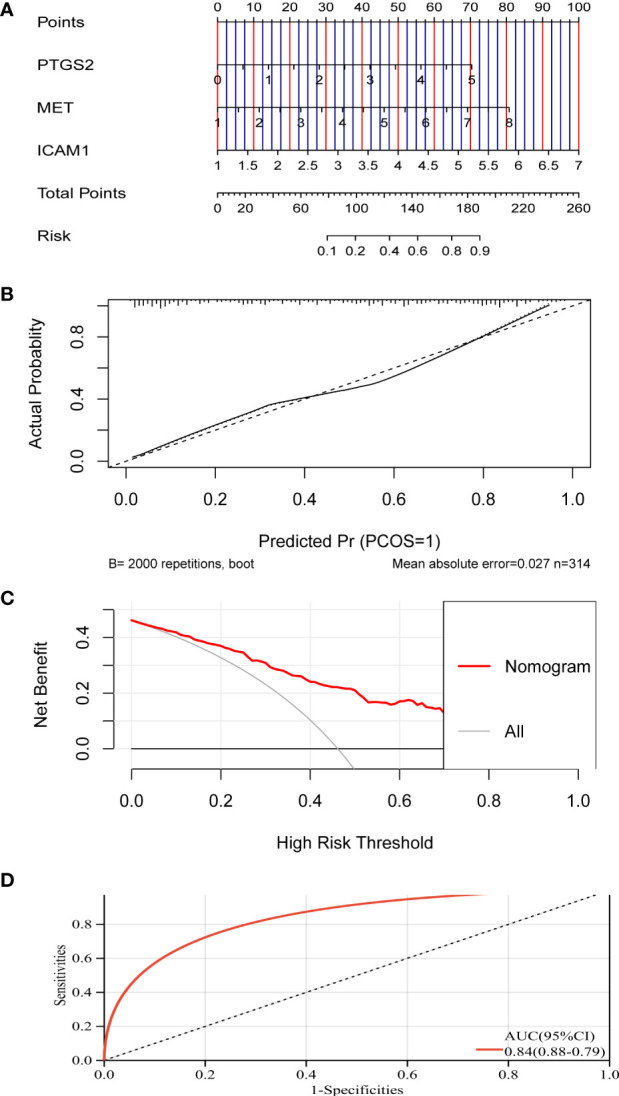
A model for predicting lymphatic metastasis of PTC based on three hub genes. **(A)** Diagnostic nomogram. **(B)** Calibration curve. **(C)** Clinical decision curve. **(D)** ROC curve based on riskscore.

As shown in **Figures 5A, B**, five genes were obtained as characteristics. In the RF model, the mean decrease in Gini values of the top five genes ranked at the median was called trait genes. Four genes namely, *MET*, *ICAM1*, *PTGS2*, and *CXCL2*, were selected based on the intersection of the two results. There was no significant difference in the expression of *CXCL2* in the process of building the logistic model. Therefore, *MET*, *ICAM1*, and *PTGS2* were selected for modeling. As depicted in **Figures 6A–D**, the nomogram was built, which had a good predictive capability for lymphatic metastasis. The AUC reached 0.84. Further, the average AUC of the model reached 0.84 after cross-validation (**Supplementary Table 1**).

### 2.5 Association between hub genes and key immune cells

**Figure 7:**
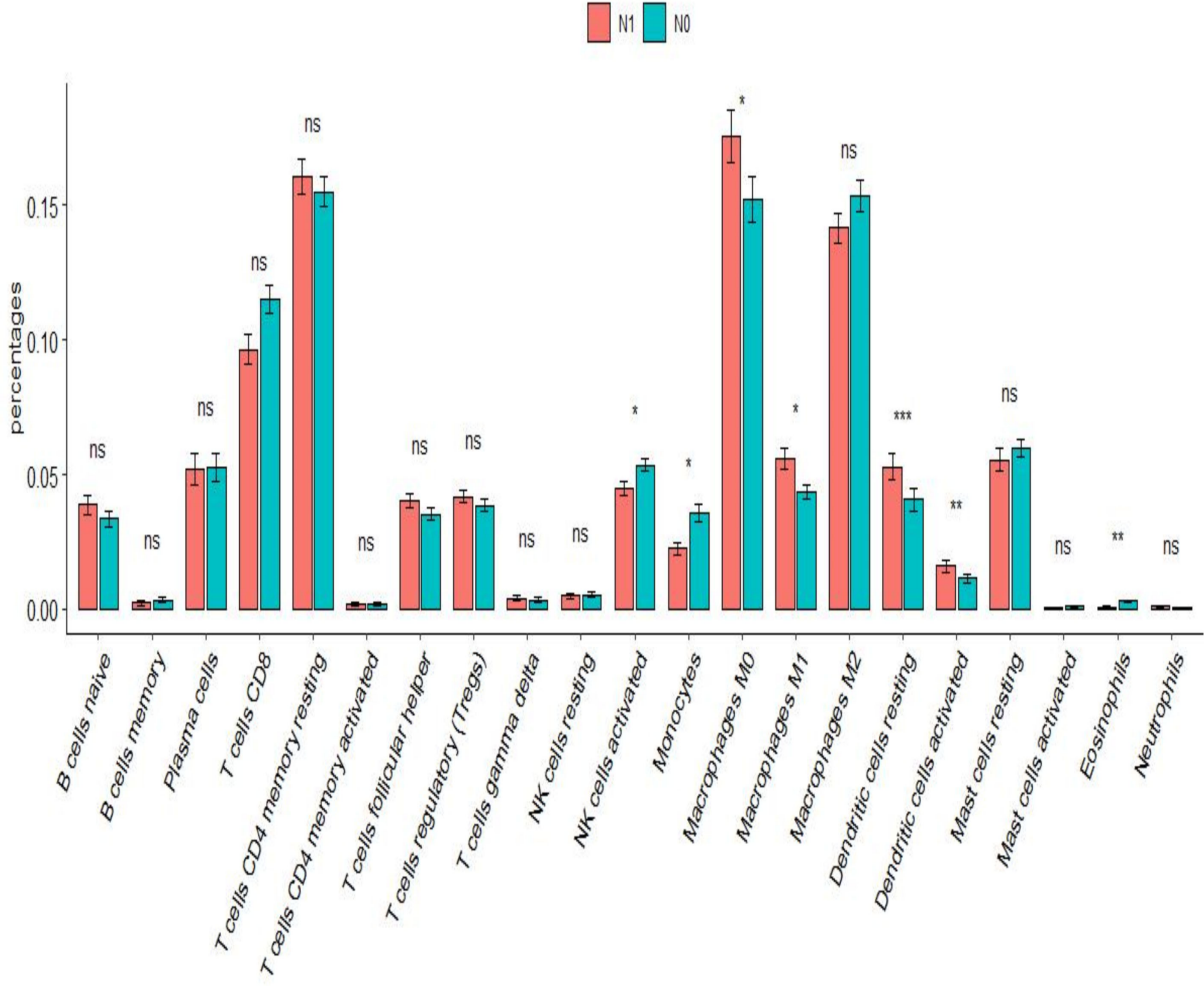
Immune infiltration analysis. Difference in proportion of immune cells between N0 and N1. *P<0.01, **P<0.001, ***P<0.0001; ns P>0.05.

**Figure 8:**
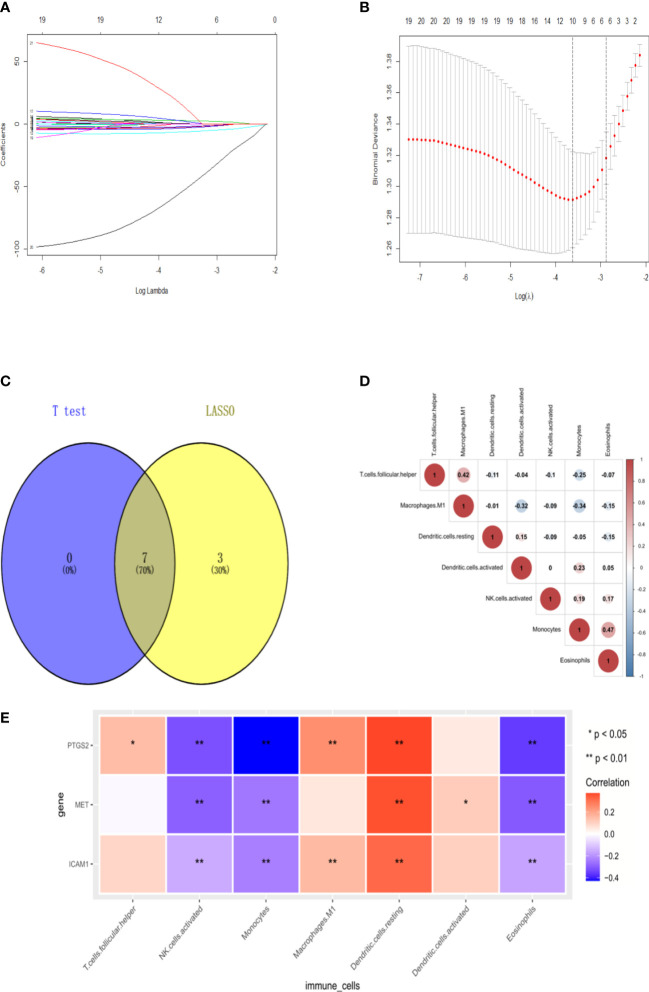
Signature immune cells. **(A-B)** Lasso regression. **(C)** Venn of common immune cells. **(D)** The heat map of Immune cell correlation. **(E)** The heat map of correlation between 3hubgenes and 7 signature immune cells.

After analysis using the R package CIBERSORT, the percentage of 22 immune cells in the N0 and N1 samples was evaluated. There was a significant difference between lymphatic metastases and non-metastases in the seven immune cells using the *t*-test (**Figure 7**). *Via* LASSO regression screening, we obtained 10 immune cells (**Figures 8A, B**). Seven immune cells, including activated NK cells, monocytes, macrophage M0 and M1, resting and activated dendritic cells, and eosinophils, were identified as key immune cells for lymphatic metastasis (**Figure 8C**). **Figure 8D** shows the correlation of the Person in the seven immune cells. As depicted in **Figure 7**, all hub genes were positively correlated with activated NK cells, monocytes, M0 and M1 macrophages, resting and activated dendritic cells, and eosinophils.

### 2.6 The results of expression differences and survival analysis

**Figure 9:**
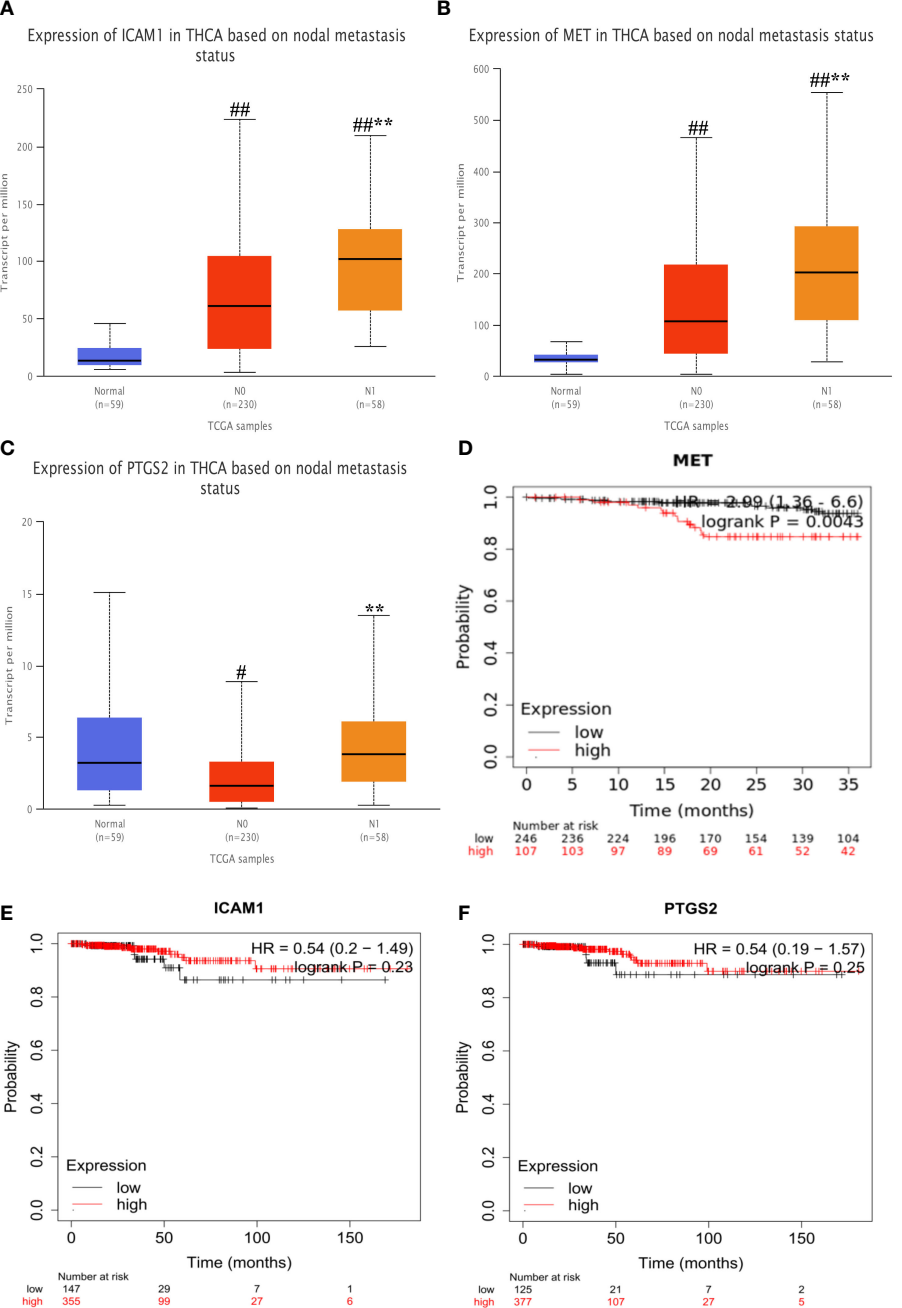
Expression differences and survival analysis in hub genes. **(A–C)** Differential expression of MET, ICAM1 and PTGS2 at different stages. **(D–F)** Survival analysis of each hub gene. Vs normal #P<0.05, ##P<0.01; vs N0**P<0.01. #P<0.05, ##P<0.01; **P<0.01. .

As shown in **Figures 9A–C**, The expression of the MET and ICAM1 in the lymphatic metastasis group were significantly higher than that in the non-metastatic group and the normal group (P<0.001). The expression of PTGS2 in the lymphatic metastasis group was significantly higher than that in the non-metastasis group (P<0.01). As shown in **Figures 9D, E**, survival analysis shows high expression of MET was strongly associated with increased risk of death in PTC patients (HR=2.09, logrank P=0.0043). However, there was no significant difference in survival time between the high and low expression groups for ICAM1 and PTGS2.

### 2.7 IHC results

**Figure 10:**
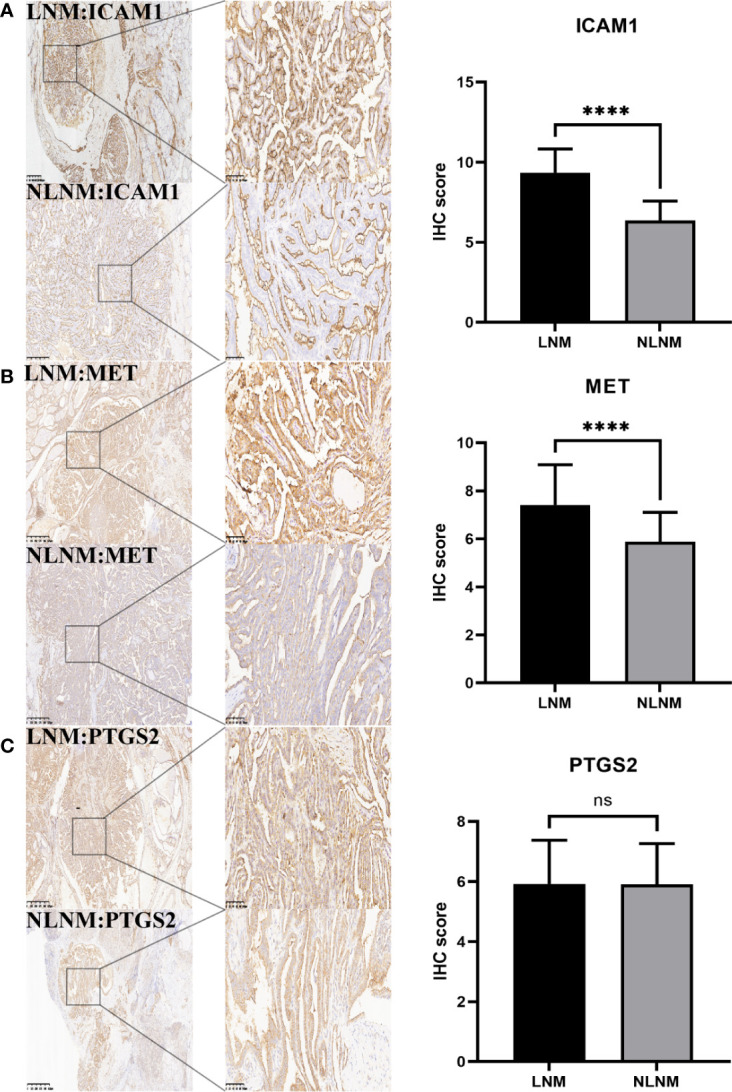
Immunohistochemical analysis. **(A)** showed that ICAM1 expression was significantly different between PTC group with lymph node metastasis and group without lymph node metastasis. **(B)** The expression of MET was significantly different in the PTC group with and without lymph node metastasis. **(C)** There was no significant difference in the expression of PTGS2 between the PTC group with and without lymph node metastasis. The IHC score was defined as the product of staining intensity (0-3) and the proportion of stained cells (0-4). ****P<0.0001.

For validation at the experimental level, the expression of PTGS2, MET, and ICAM1 was analyzed *via* immunohistochemical staining of pathological sections from the lymph node metastasis and non-metastasis groups. Results showed that there was no significant difference in the expression of PTGS2 in the two groups. MET and ICAM1 had strong staining in the lymph node metastasis group and weak staining in the lymph node non-metastasis group. According to the IHC staining results, the lymph node metastasis group had a higher expression of MET and ICAM1 than the lymph node non-metastasis group. However, the expression of PTGS2 did not significantly differ between the two groups (**Figures 10A–C**). According to Spearman correlation analysis, the expression of ICAM1 was positively correlated with the expression of MET in the PTC lymph node metastasis group (r = 0.5736, P < 0.0001, **Table 1**).

**Table 1:** The table shows the relationship between ICAM1 and MET expression in LNM.

MET	N	ICAM1		Correlation	P-value
		(+++~++++)	(+~++)		
(+++~++++)	84	82	2	0.5736	<0.0001
(+~++)	28	22	6		
Total	112	104	8

## 3 Discussion

It is a common phenomenon that lymphatic metastasis is discovered, which is one of the most close factors of tumor progression leading to a worse prognosis (5). In cases of metastasis, the lymph nodes near the primary tumor are usually affected, and lymphatic metastasis is involved in distant metastasis (6). The 5-year survival time after thyroid cancer surgery is satisfactory. Nevertheless, there is no comprehensive research on early lymphatic metastasis and regional local recurrence in PTC, and the study results are contrasting (7). Hence, biomarkers for predicting early metastasis in PTC should be identified. The current study confirmed that three hub genes, including MET, ICAM1, and PTGS2, were closely related to PTC metastasis *via* bioinformatics analysis. However, IHC showed that the expression of MET and ICAM1 significantly differed between the lymph node metastasis and non-metastasis groups. Meanwhile, there was no significant difference in the expression of PTGS2 between the two groups.

MET is located in the long arm of human chromosome No.7, and encoding hepatocyte growth factor with tyrosine kinase activity is involved in cell growth. After Met and HGF conjugation, complex self-phosphorylation activates the PI3K/AKT and Ras-MAPK signaling pathways (8). A series of studies have shown that the overexpression of the MET protein can increase the motility and invasiveness of thyroid cancer cells, leading to early lymphatic metastasis (9, 10). *ICAM-1* regulates the ICAM-1 protein belonging to the immune super protein family, which plays an important role in the process of inflammation and immunity. The overexpression of ICAM1 was linked to extrathyroidal invasion and lymph metastasis (11). Another study showed that ICAM1 was positively correlated with papillary carcinoma, but not with other types (12). Therefore, ICAM1 may be a characteristic factor of lymphatic metastasis in PTC. PTGS2 regulates the synthesis of cyclooxygenase 2, which is a key enzyme in arachidonic acid biosynthesis. In previous studies, the expression of COX-2 is upregulated in several malignant tumors, such as breast cancer and colon cancer. The overexpression of COX-2 is related to the stimulation of angiogenesis and proliferation by prostaglandins, which can promote the development of cell invasion and metastasis (13, 14). Previous studies have shown that COX-2 is overexpressed in PTC and is closely correlated with tumor invasiveness (15, 16).

The lymphatic system participates in immune regulation. Tumor lymph node metastasis is usually related to this process *via* immune infiltration (17). In this immune infiltration analysis, the three hub genes were closely correlated with activated NK cells, monocytes, resting dendritic cells, and eosinophils. For example, the proportion of dendritic and resting dendritic cells in metastatic samples increased significantly. Previous studies have revealed that immature dendritic and dendritic cells were highly expressed in patients with PTC (18, 19). Various degrees of inflammation are typically correlated with tumor development, and the expression of ICAM-1 in the lymphatic vessels is upregulated during inflammation. Thus, the association between the expression of Mac-1 and ICAM-1 in dendritic cells and inflamed lymphatic endothelium, respectively, may decrease the ability of dendritic cells to activate T cells (20). The IRGs derived in this study might contribute to the progression of lymph node metastasis in PTC by triggering tumor-associated immune infiltration. In this paper, we construct a prediction model innovatively, which makes up for the shortcomings of relevant papers. However, the results of IHC verification and bioinformatics analysis of differences in the expression of PTGS2 are not consistent. Thus, this must be studied further. In addition, the experimental verification part of this study is not sufficient, which can’t well verify the conclusions of the paper. Therefore, more perfect experimental verification is needed in the future.

## 4 Methods and materials

### 4.1 Data processing

**Figure 11:**
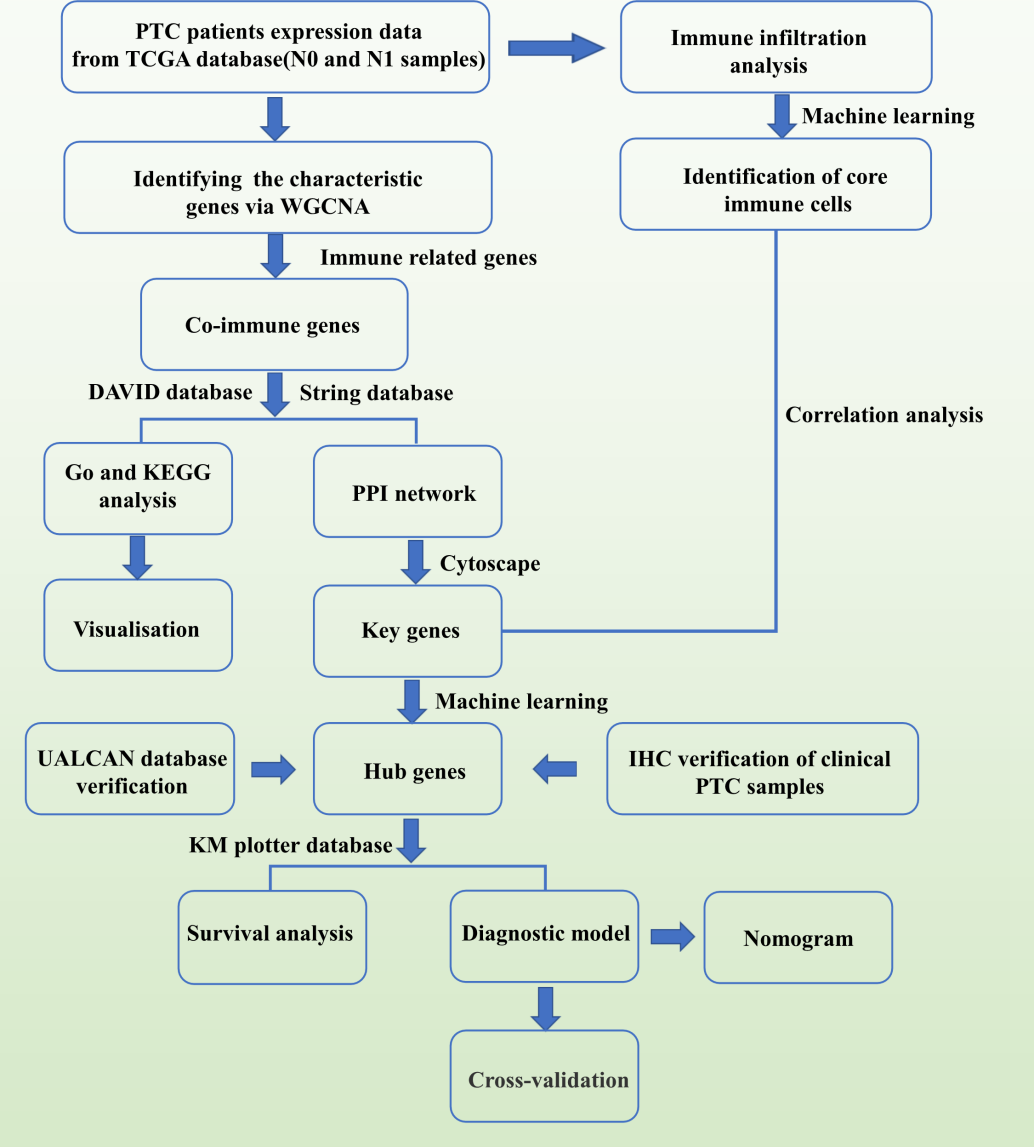
Flowchart related to this study.

**Figure 11** depicts the flow chart of data collection, processing, analysis, and validation. The PTC datasets were downloaded from The Cancer Genome Atlas database. Further, they included 145 samples with lymphatic metastases (N1, N1a, and N1b) and 169 samples without lymphatic metastases (N0). Then, the gene expression matrix was extracted.

### 4.2 Establishment of the co-expression network

Weighted correlation network analysis was performed using an R package to construct the gene co-expression network (21). Then, an adjacency matrix was established to evaluate the correlation between the strengths of nodes. In this study, the optimal soft threshold β was set to 10 and the scale-free R2 to 0.9. Then, the adjacency matrix was converted to a topological overlap matrix. Hierarchical clustering was used to distinguish modules, each of which included at least 50 genes. Eigengene was calculated, and similar modules were merged (abline = 0.3). Finally, the hierarchical clustering tree of genes was established using the hierarchical clustering method. Closely related co-expressed gene modules were identified using the dynamic tree-cutting method, and the correlation between module signature genes and clinical features was analyzed using Pearson’s correlation coefficient. Pearson’s correlation tests were used to analyze the relationship between HCC and modules, with normal persons and cancer patients assigned values of 0 and 1, respectively. The module eigengene (ME) indicates the gene expression value shared by all modules. The MM value represents the relationship between ME and the gene expression profile (MMi = cor (x(i)), ME; where i is the value of each gene). The closer a gene’s MM value is to 1, the more essential that gene is in a certain gene module. The gene significance (GS) value reflects the relationship between HCC and the genes (GS = log(p); where p is the Student’s t-test value). The intramodular connectivity (K.in) score was the average of all connections between genes inside a module.

### 4.3 Enrichment analysis and identification of key immune genes

The genes in the module with the highest correlation to the N1 phase were screened and intersected with immune genes in the Immport database. Subsequently, these co-IRGs underwent Gene Ontology (GO) and Kyoto Encyclopedia of Genes and Genomes (KEGG) analysis using the R package clusterProfiler. They were imported into the String database to construct the protein–protein interaction (PPI) network. Then, the top 10 key IRGs were obtained using the cytoHubba application in Cytoscape.

### 4.4 Prognostic modelling

Ten key IRGs correlated with lymphatic metastasis were entered into the LASSO regression and RF models to screen out the hub genes. The data is divided into two parts according to 60%:40%. 60% was established using the logistic regression model. 40% was validation set Moreover, a diagnostic nomogram, calibration curve, and clinical decision curve were drawn. Finally, A 10-fold and 200-times internal cross-validation strategy was used, and this process was repeated 2000 times to acquire area under the curve (AUC) for evaluating model accuracy.

### 4.5 Immune infiltration assessment

Expression profile data were used in immune infiltration analysis using the R package CIBERSORT (22). Differential immune cells between N1 and N0 were obtained using the *t*-test, and characteristic immune genes were obtained using the RF model. The intersection of the abovementioned results was used as key immune cells in this study. Spearman’s correlation method was used to analyze key immune cells and hub genes.

### 4.6 Expression differences and survival analysis in hub genes

The expression of the three hub genes in different stages of thyroid cancer was retrieved separately in the UALCAN database (https://ualcan.path.uab.edu/) for comparison between groups. Retrieval of the expression of the three hub genes in relation to survival time of thyroid cancer patients in KM plotter database (http://kmplot.com/analysis/).

### 4.7 Clinical sample collection

Data on 224 clinical cases of PTC diagnosed based on pathological examination results after thyroidectomy and lymph node dissection in the neck were collected from October 2020 to October 2022 at the Department of General Surgery, Baoding First Central Hospital. All PTC tissue specimens were selected from wax blocks embedded in the pathology department of the First Central Hospital of Baoding. In total, 112 cases involved lymph node metastases (≥ 5) and 112 did not. Among them, the lymph node metastasis group: 112 cases (100%) in T1N1M0 and the lymph node non-metastasis group: 112 cases (100%) in T1N0M0. TNM staging was performed according to the 2018 version of the American Joint Anti-Cancer Consortium staging. All patients initially underwent surgery. However, they did not receive preoperative radiotherapy and chemotherapy and were pathologically diagnosed by two senior pathologists.

### 4.8 Immunohistochemistry and image analysis

Tissue wax blocks were serially sectioned at 4-μm thickness and placed in a 68°C baker for 25 min. The sections were soaked in xylene for 40 min and in anhydrous ethanol for 20 min. After rinsing with PBS, the sections were heated in citrate buffer for 15 min., cooled, and rinsed using PBS. Three sections were taken from the same patient and incubated dropwise with anti-PTGS2 antibodies (PBS1:100 dilution), anti-MET antibodies (PBS1:100 dilution), and anti-ICAM1 antibodies (PBS1:100 dilution). Goat anti-mouse/rabbit IgG polymer antibody was then added and incubated. Negative controls were prepared using PBS instead of primary antibody. After PBS rinsing, they were incubated with diaminobenzidine for 5 min. Finally, the slices were dehydrated using alcohol and xylene and sealed. The area of positive cells: < 5% cell staining is 0 points; 6% ~ 25% cells staining is 1 points; 26% ~ 50% cells staining is 2 points; 51% ~ 75% cells staining is 3 points; 76% ~ 100% cell staining is 4 points. Positive cells of tinting strength: -: 0 points; +:1 points; ++: 2 points; +++: 3 points. The immunohistochemistry (IHC) score was defined as the product of staining intensity (0–3) and the proportion of stained cells (0–4). Final results: 0-4 was low expression (+~++), 6-12 was high expression (+++~++++).

### 4.9 Statistical analysis

The two-tailed Student’s *t-*test was used to analyze the statistical significance of ICAM1, MET, and PTGS2 expression levels and IHC scores between lymph node metastatic and non-metastatic tissues in patients with papillary thyroid carcinoma. A P value of < 0.05 was considered statistically significant. Spearman analysis was performed to evaluate the correlation between the expression levels of ICAM1, MET, and PTGS2 and the level of immune cell infiltration. The correlations among ICAM1, MET, and PTGS2 expression levels were assessed *via* Spearman analysis.

## 5 Conclusion

Via bioinformatics analysis and experimental validation, the expressions of MET and ICAM1 were found to be upregulated in patients with lymph node metastasis from papillary thyroid carcinoma. Further, two hub genes were closely correlated with activated NK cells, monocytes, resting dendritic cells, and eosinophils. Thus, these genes can be novel molecular biomarkers and therapeutic targets in lymph node metastasis from papillary thyroid carcinoma.

## Data availability statement

The original contributions presented in the study are included in the article/**Supplementary Material**. Further inquiries can be directed to the corresponding author.

## Ethics statement

The studies involving human participants were reviewed and approved by Institutional Ethics Committee of the First Central Hospital of Baoding. The patients/participants provided their written informed consent to participate in this study. Written informed consent was obtained from the individual(s) for the publication of any potentially identifiable images or data included in this article.

## Author contributions

Designed and supervised the study: YY, XG, and HZ. Acquisition of data: YY, YJ, JC and JS. Drafting of the manuscript: YY, XG. All authors contributed to the article and approved the submitted version.
